# Epidemiology of Major Bacterial Pathogens Associated with Porcine Respiratory Disease Complex: A Cross-Sectional Study from Intensive Swine Farms in Xinjiang, China (2024–2025)

**DOI:** 10.3390/vetsci13040366

**Published:** 2026-04-09

**Authors:** Yaqi Guo, Yanfang Li, Zhenglong Wen, Yan Liang, Kexun Lian, Pei Zheng, Yonggang Qu

**Affiliations:** 1 College of Animal Science and Technology, Shihezi University, Shihezi 832003, China; guoyaqi@stu.shzu.edu.cn (Y.G.);; 2 Xinjiang Tecon Animal Husbandry Technology Co., Ltd., Changji 831399, China

**Keywords:** *Glaesserella parasuis* (HPS), *Actinobacillus pleuropneumoniae* (APP), *Streptococcus suis* (SS), *Pasteurella multocida* (PM), serotype distribution, co-infection

## Abstract

*Glaesserella parasuis* (formerly *Haemophilus parasuis*, HPS), *Actinobacillus pleuropneumoniae* (APP), *Streptococcus suis* (SS), and *Pasteurella multocida* (PM) are common bacterial pathogens associated with Porcine Respiratory Disease Complex (PRDC), a major cause of economic losses in the swine industry. To address this, a cross-sectional study was conducted across 27 large-scale swine farms in Xinjiang, China (October 2024–May 2025). A total of 1239 clinical samples were analyzed by species-specific PCR, and positive samples were further serotyped. Overall, SS and HPS were the predominant pathogens, with higher detection rates in winter and spring. Notably, SS and HPS were most frequent in nasal swabs, while APP and PM predominated in tissue samples. Furthermore, co-infections were common, with HPS + SS being the most prevalent. Serotyping revealed dominance of HPS serotype 12, APP serotype 12, SS serotype 3, and PM serotypes A and B (serotypes E and F not detected). In addition, SS was also detected in environmental samples and farm workers’ nasal swabs. These findings suggest that future prevention and control strategies should focus on developing multivalent vaccines targeting the predominant serotypes identified, implementing regular serotype surveillance to guide precision immunization protocols, and strengthening environmental disinfection and biosecurity practices to reduce co-infections and occupational exposure risks.

## Simple Summary

Porcine Respiratory Disease Complex, driven by bacterial co-infections, causes major economic losses in intensive swine production. This study investigated four key pathogens associated with this condition—*Glaesserella parasuis*, *Actinobacillus pleuropneumoniae*, *Streptococcus suis*, and *Pasteurella multocida*—across 27 large-scale pig farms in Xinjiang, China (October 2024–May 2025). *Streptococcus suis* and *Glaesserella parasuis* were the predominant pathogens, with peak detection in winter and spring. Co-infections (especially *Glaesserella parasuis* +*Streptococcus suis*) were frequent. Serotype diversity was high: *Glaesserella parasuis* serotype 12, *Actinobacillus pleuropneumoniae* serotype 12, *Streptococcus suis* serotype 3, and *Pasteurella multocida* serotypes A and B dominated. Notably, *Streptococcus suis* was detected in farm environments and workers’ nasal swabs, indicating potential zoonotic risks. These findings highlight the need for enhanced surveillance and biosecurity to control Porcine Respiratory Disease Complex in large-scale farms.

## 1. Introduction

Porcine Respiratory Disease Complex (PRDC), first described in 1998, refers to a multifactorial respiratory condition in pigs arising from the interplay of environmental conditions, management practices, population size, age, and genetic factors [1,2,3]. PRDC imposes substantial economic burdens on global pig production, driven by high morbidity, reduced weight gain, increased culling rates, and elevated medication and labor costs [4]. The disease predominantly affects pigs aged 14 to 22 weeks [2,5], with morbidity typically ranging from 35% to 70% and mortality reaching 14% to 35% [6].

Xinjiang is one of China’s key pig-producing regions, hosting numerous large-scale farms across its diverse geographical and climatic zones [7]. However, the region’s unique environmental characteristics: including prolonged cold winters, marked diurnal temperature fluctuations, and limited ventilation in enclosed pig houses during cold seasons—may facilitate the transmission and spread of respiratory pathogens, exacerbating disease prevalence [8].

The etiology of PRDC is inherently complex. Among bacterial agents, a core group of bacteria (e.g., *Actinobacillus pleuropneumoniae*, APP) can independently cause disease, while others such as *Glaesserella parasuis* (formerly *Haemophilus parasuis*, HPS), *Streptococcus suis* (SS), and *Pasteurella multocida* (PM) frequently act as secondary pathogens, exacerbating clinical [9,10,11,12]. APP and HPS, members of the *Pasteurellaceae* family, are associated with various systemic diseases, including polyserositis, pleuritis, meningitis, arthritis, and acute pneumonia [13]. PM is a commensal bacterium that can opportunistically act as a secondary pathogen during respiratory infections initiated by other agents, such as *Mycoplasma hyopneumoniae* [14]. SS, a Gram-positive bacterium, primarily causes septicemia but also contributes as a secondary pathogen in PRDC [11].

When pigs are co-infected with two or more respiratory pathogens, the interactions at the cellular and molecular levels become exceedingly complex. Bacteria such as HPS and SS colonize pigs early in life, and subclinically infected pigs can serve as reservoirs for transmission to susceptible cohorts. These interactions involve multiple biological mechanisms, including pathogen competition and synergy, as well as modulation of host immune responses. Confronted with mixed infections, the challenge becomes multifaceted and multifactorial [15]. Co-infections in PRDC are more prevalent on farms than single infections, and their pathogenicity is not merely additive but exacerbates disease severity through a chain reaction involving “immunosuppression—barrier disruption—enhanced inflammation” [16]. To date, the efficacy of vaccines against these bacterial pathogens has been inconsistent, largely due to serotypic diversity, poor cross-protection, limited protective efficacy, and interference from maternally derived antibodies.

Early detection of PRDC is crucial for enabling timely interventions at the farm level, thereby preventing and minimizing potential losses. Recent advances in artificial intelligence (AI) have shown promise for early detection and monitoring of PRDC [17]. Nevertheless, current technologies remain constrained by certain limitations, and substantial efforts are required to overcome these technical bottlenecks to develop more intelligent AI technologies for monitoring porcine respiratory health.

However, epidemiological data on these bacterial pathogens remain limited in Xinjiang, and their serotype profiles in this region have not been systematically characterized. Given the region’s unique environmental conditions and the critical role of serotype diversity in vaccine efficacy, understanding the local epidemiological landscape is essential for developing targeted control strategies. Beyond its local importance, PRDC is a global challenge, and the epidemiological patterns of these bacterial pathogens can vary considerably across regions. Therefore, systematic investigations in distinct production systems are needed to inform region-specific interventions and contribute to the broader understanding of PRDC epidemiology worldwide. Based on these considerations, we hypothesized that the prevalence and serotype distribution of these four pathogens would vary across farms and seasons, and that some of them may pose zoonotic risks. To test this hypothesis, this study aimed to investigate the prevalence, co-infection patterns, and serotype distribution of these four major PRDC-associated bacterial pathogens in large-scale swine farms across Xinjiang.

## 2. Materials and Methods

### 2.1. Sample Collection

**Figure 1:**
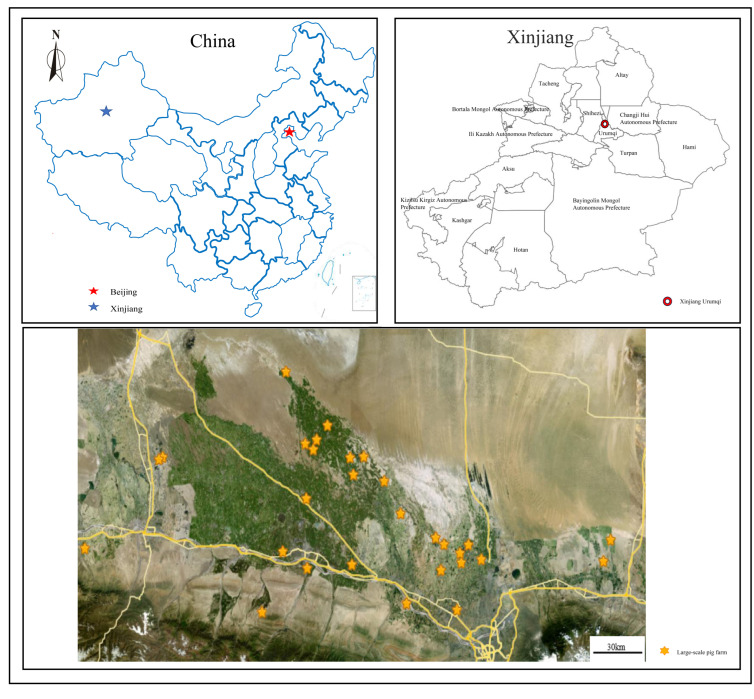
Geographical distribution of sampling sites in Xinjiang, China. The map illustrates the locations of 27 large-scale swine farms from which clinical, abattoir, and environmental samples were collected between October 2024 and May 2025.

**Figure 2:**
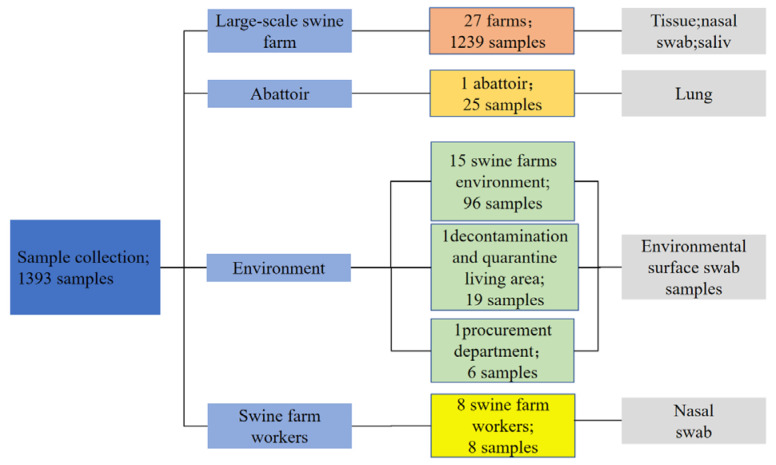
Sample collection sources and quantities. A total of 1393 samples were collected from 27 large-scale swine farms, one abattoir, farm environments, decontamination and quarantine areas, procurement departments, and swine farm workers across Xinjiang, China, between October 2024 and May 2025. Sample types included tissue, nasal swabs, saliva, lung tissue, and environmental surface swabs.

From October 2024 to May 2025, a total of 1393 samples were collected across Xinjiang, China, employing a multi-point surveillance strategy to investigate the prevalence and epidemiological characteristics of HPS, APP, SS, and PM. Samples were obtained from clinical cases on farms, abattoir, and environmental sources. The sampling sites were distributed across multiple locations in Xinjiang, including Changji City, Shihezi City, Wusu City, Fukang City, Huyanghe City, Shawan City, Miquan City, Hutubi County, Manas County, and Wujiaqu. All samples were transported to the laboratory under refrigerated conditions and processed within 24 h of collection. The geographical distribution of sampling sites is presented in Figure 1, and sample collection sources and quantities in Figure 2. Detailed information is provided in Table A2.

#### 2.1.1. Clinical Samples from Farms Cases

**Figure 3:**
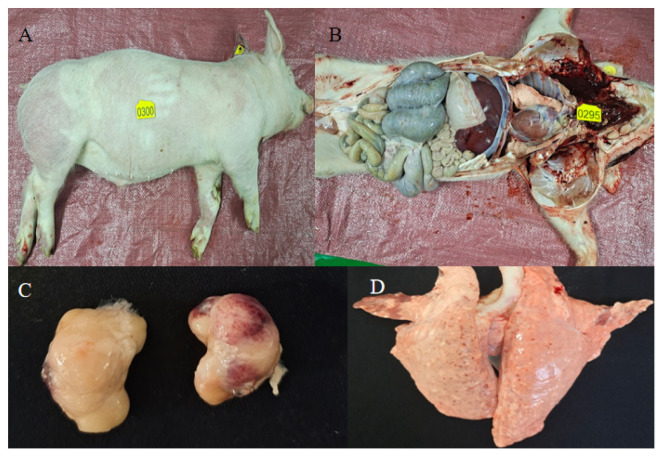
Pathological findings in pigs that succumbed to respiratory disease. (**A**) Gross appearance of the carcass: purple ecchymoses and petechiae distributed on the ears, neck, ventral abdomen, and limbs. (**B**) Subcutaneous tissue of a pig that died from respiratory disease. (**C**) Enlarged and congested submandibular lymph nodes, indicating regional inflammatory response. (**D**) Lung surface with multifocal red discoloration and hemorrhagic foci, suggestive of acute pneumonia.

A total of 1239 clinical samples were collected from 27 commercial pig farms. Sampling targeted pigs based on clinical presentation: nasal swabs or saliva were obtained from live animals showing respiratory signs (e.g., coughing, dyspnea), while tissue samples (lung, heart, spleen, lymph nodes, tonsils) were collected during necropsy from pigs that died suddenly, following standard diagnostic procedures [18] (Figure 3). This included 203 nasal swabs, 115 saliva samples, and 341 tissue samples. Additionally, during targeted surveys in December 2024 and March 2025, 580 samples were collected from pigs displaying various clinical abnormalities (e.g., fever, diarrhea, neurological signs) for HPS, APP, SS, and PM screening.

#### 2.1.2. Tissue Samples from Abattoir

To assess the disease status of slaughtered pigs, 25 fresh lung samples with gross pathological lesions were collected at an abattoir in Wujiaqu, as described in previous studies [19,20,21].

#### 2.1.3. Environmental and Personnel-Associated Samples

Environmental surveillance included 96 surface swab samples collected from the animal housing and common areas of 15 pig farms, following established protocols [22]. Personnel surveillance comprised 8 nasal swabs collected from farm workers after their shift at 8 of these farms. Beyond the farm premises, 19 environmental swabs were obtained from a centralized disinfection and isolation dormitory, and 6 samples were collected from a shared logistics area handling vehicles, personnel, supplies. In total, 129 environmental and human-associated samples were included in the analysis.

### 2.2. Sample Processing and Nucleic Acid Extraction

Total nucleic acids were extracted from all samples using an automated nucleic acid platform (Beijing Biaochi Zehui Biotechnology Co., Ltd., Beijing, China) with the Rapid Magnetic Bead Method Virus DNA/RNA Extraction Kit (Advancing and Devoting Biotechnology Co., Ltd., Beijing, China). For nasal swabs and saliva samples were processed directly by taking 300 µL of the vortex-mixed liquid. For tissue samples, a visibly lesioned portion (approximately 1.0 g) was aseptically excised after rinsing the surface with sterile saline. The tissue was homogenized in saline using a tissue homogenizer (Ningbo Xinzhi Biotechnology Co., Ltd., Ningbo, China) (65 Hz, 400 s), and the homogenate was centrifuged at 4 °C. A 300 µL aliquot of the supernatant was then used for extraction. Extracted nucleic acids were stored at −20 °C until further analysis.

### 2.3. PCR Detection and Serotyping

Species-specific primers for identification of HPS, APP, SS, and PM were synthesized. Considering the extensive serotypic diversity of HPS, APP, and SS, a panel of primers targeting prevalent and highly virulent serotypes—based on both literature reports and our preliminary regional investigations—was designed for PCR-based serotyping [6,23,24]. Specifically, we targeted HPS serotypes 10, 12, 13, and 15; APP serotypes 1, 2, 7, 12, and 15; SS serotypes 1, 2, 3, 7, 9, 8, and 18; and PM capsular types A, B, D, E, and F. All primers were synthesized by Youkang Biotechnology Co., Ltd. (Xinjiang, China), and detailed primer sequences are provided in Table A1. Positive control plasmids were purchased from Sangon Biotech Co., Ltd. (Shanghai, China).

All PCR amplifications were performed in 25 μL reaction mixtures containing 12.5 μL of 2× EasyTaq PCR Super Mix (NanJing Vazyme Biotech Co., Ltd., Nanjing, China), 0.5 μL each of forward and reverse primer, 1 μL of DNA template, and nuclease-free water to adjust the final volume. The thermal cycling conditions were as follows: initial denaturation at 94 °C for 5 min, followed by 35 cycles of 94 °C for 30 s, 56 °C for 30 s, and 72 °C for 90 s, with a final extension at 72 °C for 10 min. Amplification products were analyzed by electrophoresis on 1% agarose gels(Beijing Junyi Dongfang Electrophoresis Equipment Co., Ltd., Beijing, China) and visualized under UV light.

### 2.4. Statistical Analysis

All statistical analyses were performed using GraphPad Prism (version 9.0, GraphPad Software, San Diego, CA, USA) and Origin (version 2025, OriginLab Corporation, Northampton, MA, USA). Descriptive statistics were used to calculate pathogen detection rates and serotype distributions. Comparisons of detection rates among different sample types, seasons, and farms were analyzed using the chi-square test or Fisher’s exact test, as appropriate. *p* < 0.05 was considered statistically significant. All figures were generated using the same software packages.

## 3. Results

### 3.1. Prevalence of the Four Major Bacterial Pathogens

**Figure 4:**
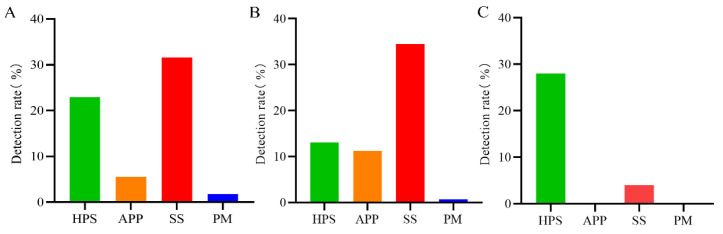
Detection rates of HPS, APP, SS, and PM in different sample populations from swine farms and an abattoir in Xinjiang. (**A**) Overall detection rates among 1239 clinical samples collected from 27 large-scale farms. (**B**) Detection rates among 580 samples collected from pigs exhibiting clinical abnormalities (e.g., fever, respiratory distress, neurological signs). (**C**) Detection rates in 25 grossly abnormal lung tissue samples collected from slaughtered pigs at an abattoir in Wujiaqu.

From October 2024 to May 2025, a total of 1239 clinical samples were collected to assess the prevalence of four major bacterial respiratory pathogens. The overall detection rates are shown in Figure 4A. A chi-square test revealed significant differences among the four pathogens (χ^2^ = 503.1, *p* < 0.001). SS exhibited the highest detection rate at 32.12% (398/1239), followed by HPS at 22.84% (283/1239). In contrast, the detection rates of APP and PM were relatively lower, at 5.65% (70/1239) and 1.78% (22/1239), respectively. Pairwise comparisons using the Bonferroni correction (α′ = 0.0083) showed that the detection rate of SS was significantly higher than those of HPS, APP, and PM (all *p* < 0.001); the detection rate of HPS was significantly higher than those of APP and PM (both *p* < 0.001); and the detection rate of APP was also significantly higher than that of PM (*p* < 0.001). These results indicate that SS and HPS are the predominant bacterial pathogens among the four species investigated in the Xinjiang.

Further analysis was conducted on 580 samples collected from clinically abnormal pigs (Figure 4B). Consistent with the overall findings, the detection rates of the four pathogens differed significantly (χ^2^ = 286.5, *p* < 0.001). Multiple comparisons with Bonferroni correction (α’ = 0.0083) revealed that the detection rate of SS (37.76%, 219/580) was significantly higher than those of HPS (25.86%, 150/580), APP (2.41%, 14/580), and PM (1.21%, 7/580) (all *p* < 0.001). The detection rate of HPS was significantly higher than those of APP and PM (both *p* < 0.001). However, no statistically significant difference was observed between the detection rates of APP and PM (*p* = 0.123). These findings further corroborate the dominant roles of SS and HPS among the four bacterial pathogens in Xinjiang, whether as primary agents responsible for respiratory pathology or as secondary pathogens requiring co-factors to precipitate disease.

Additionally, a total of 25 abnormal lung samples were collected from two batches of slaughtered pigs (Figure 4C). Among these samples, HPS was detected in 28.00% (7/25), while SS was detected in 4.00% (1/25). Neither APP nor PM was detected in these samples.

### 3.2. Co-Infection Patterns of the Four Major Bacterial Pathogens

**Figure 5:**
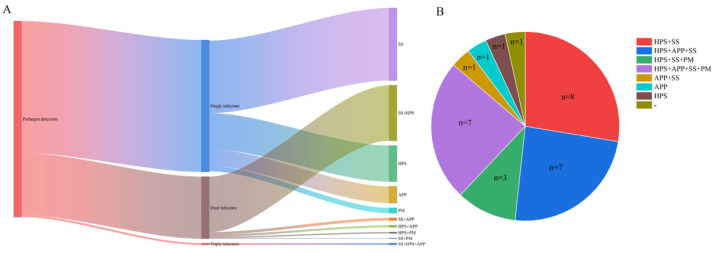
Co-infection patterns of HPS, APP, SS, and PM in positive samples and across farms. (**A**) Distribution of mono-infections, dual-infections, and triple-infections among 583 positive samples. Dual-infections include HPS + SS, APP + SS, APP + HPS, HPS + PM, and PM + SS; triple-infections were observed only as APP + HPS + SS. (**B**) Pathogen detection profiles across 27 individual farms, ranging from no pathogen detected to quadruple-infections involving all four pathogens.

A total of 583 positive samples with detectable HPS, APP, SS, or PM were analyzed for co-infection patterns (Figure 5A). Among these, mono-infections accounted for 67.07% of positive samples, with SS representing the highest proportion at 37.22%, followed by HPS at 18.52%, APP at 8.58%, and PM at 2.74%. Dual-infections comprised 31.90% of positive samples, which HPS + SS being the most prevalent combination at 28.47%, followed by APP + SS at 1.37%, APP + HPS at 1.03%, HPS + PM at 0.69%, and PM + SS at 0.34%. Triple-infections were observed only as the combination of APP + HPS + SS, accounting for 1.03% of positive samples. No quadruple-infections were detected.

At the farm level, individual farms exhibited diverse pathogen detection profiles, ranging from no pathogen detected to co-infections involving up to four pathogens (Figure 5B). Among the 27 farms surveyed, one farm had no detectable pathogens; mono-infections were identified in two farms (one with HPS alone, one with APP alone); dual-infections were found in nine farms, including HPS + SS (n = 8) and APP + SS (n = 1); triple-infections were observed in ten farms, comprising HPS + APP + SS (n = 7) and HPS + SS + PM (n = 3); and quadruple-infections involving all four pathogens (HPS + APP + SS + PM) were detected in seven farms.

### 3.3. Detection Rates of the Four Major Bacterial Pathogens in Different Sample Types

**Figure 6:**
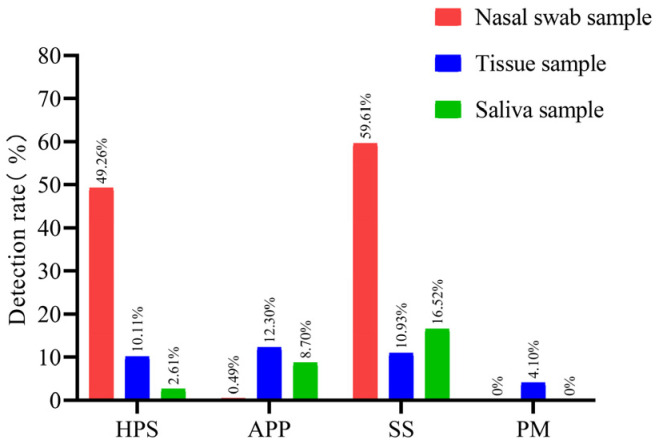
Detection rates of HPS, APP, SS, and PM in different sample types collected from large-scale swine farms in Xinjiang. Samples included nasal swabs (n = 203), saliva samples (n = 115), and tissue samples (n = 366) from clinical cases.

The detection rates of the four pathogens varied considerably across different sample types (Figure 6). HPS was most frequently detected in nasal swabs (49.26%, 100/203), followed by tissue samples (10.11%, 37/366) and saliva samples (2.61%, 3/115). Pairwise comparisons showed that the detection rate in nasal swabs was significantly higher than those in tissue and saliva samples (both *p* < 0.001). APP exhibited the highest detection rate in tissue samples (12.30%, 45/366), followed by saliva samples (8.70%, 10/115) and nasal swabs (0.49%, 1/203). The detection rates in tissue and saliva samples were significantly higher than that in nasal swabs (both *p* < 0.001), but no significant difference was observed between tissue and saliva samples (*p* = 0.12). SS was most prevalent in nasal swabs (59.61%, 121/203), followed by saliva samples (16.52%, 19/115) and tissue samples (10.93%, 40/366). The detection rate in nasal swabs was significantly higher than those in tissue and saliva samples (both *p* < 0.001), with no significant difference between saliva and tissue samples. PM was detected exclusively in tissue samples (4.10%, 15/366), and its detection rate was significantly higher than those in nasal swabs and saliva samples (both *p* < 0.001).

Within the same sample type, distinct differences were also observed among the detection rates of different pathogens. In nasal swabs, the detection rates of SS and HPS were significantly higher than those of APP and PM (all *p* < 0.001), and the detection rate of SS was slightly higher than that of HPS (*p* = 0.007). In tissue samples, no significant differences were found among the detection rates of HPS, APP, and SS (all *p* > 0.05), but all three were significantly higher than that of PM (all *p* < 0.001). In saliva samples, SS exhibited the highest detection rate, followed by APP, while HPS and PM showed relatively low rates. The detection rate of SS was significantly higher than those of HPS and PM (both *p* < 0.001), and the detection rate of APP was significantly higher than that of PM (*p* < 0.001); however, no significant difference was found between APP and HPS (*p* = 0.07).

### 3.4. Detection Rates of the Four Major Bacterial Pathogens in 2024 and 2025

**Figure 7:**
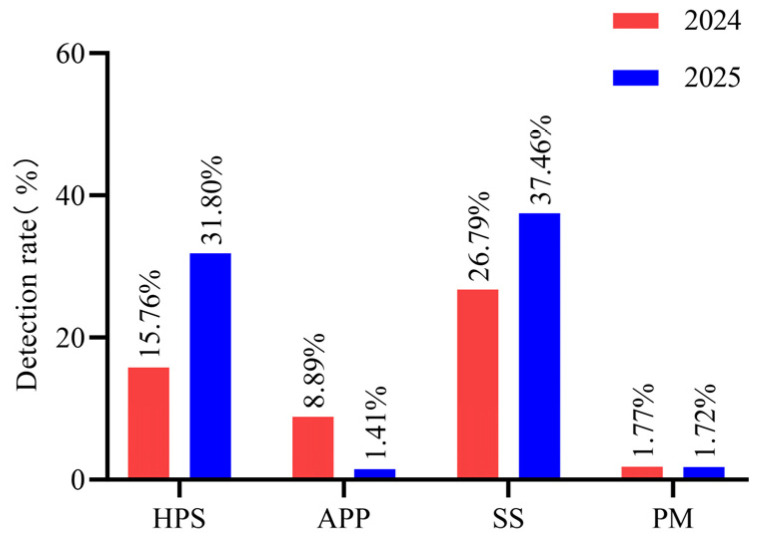
Yearly comparison of HPS, APP, SS, and PM detection rates in large-scale swine farms in Xinjiang from 2024 to 2025. Detection rates for HPS, SS, and PM increased, whereas APP detection declined sharply. HPS rose from 15.76% (110/698) to 31.80% (180/566); SS increased from 26.79% (187/698) to 37.46% (212/566); PM exhibited a slight increase from 1.72% (12/698) to 1.77% (10/566); and APP decreased from 8.89% (62/698) to 1.41% (8/566).

A total of 698 clinical samples were collected in 2024 and 566 in 2025. Comparison of detection rates between 2024 and 2025 (Figure 7) showed that the detection rate of APP decreased significantly from 8.89% (62/698) to 1.41% (8/566) (χ^2^ = 34.52, *p* < 0.001); the detection rate of HPS increased significantly from 15.76% (110/698) to 31.80% (180/566) (χ^2^ = 44.88, *p* < 0.001); and the detection rate of SS increased significantly from 26.79% (187/698) to 37.46% (212/566) (χ^2^ = 17.33, *p* < 0.001). The detection rate of PM increased slightly from 1.72% (12/698) to 1.77% (10/566), but the difference was not statistically significant (χ^2^ = 0.004, *p* = 0.949). These findings indicate that during the monitoring period, the detection rates of HPS and SS increased significantly, while that of APP decreased significantly, and PM remained at a low level with no significant change.

### 3.5. Monthly Detection Rates of the Four Major Bacterial Pathogens

**Figure 8:**
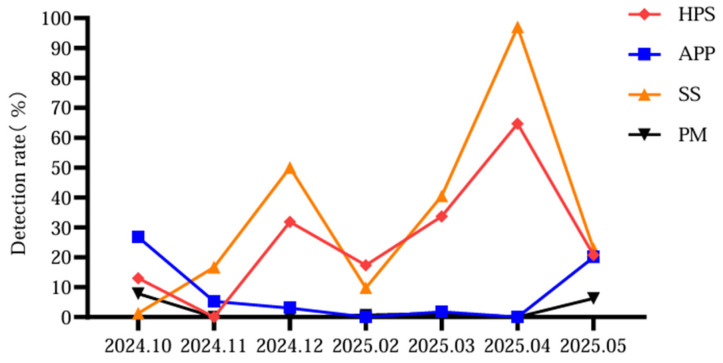
Monthly detection rates of HPS, APP, SS, and PM in large-scale swine farms in Xinjiang from October 2024 to May 2025 (No samples were collected in January 2025 due to the holiday period).

The detection rates of the four pathogens were analyzed by month (Figure 8). HPS and SS exhibited marked increase during the winter and spring months (December and April), with rates considerably higher than those observed in other months. In contrast, APP and PM showed relatively higher detection rates on October and May.

This seasonal pattern is likely attributed to the climatic characteristics of Xinjiang and associated swine farm management practices. At the onset of winter, abrupt temperature drops and large diurnal temperature variations prompt farmers to completely enclose pig houses for thermal insulation, leading to reduced ventilation and increased pathogen accumulation. During the winter-spring transition, temperatures begin to rise but remain highly unstable; such drastic climatic fluctuations impose additional physiological stress on pigs, potentially compromising their immune status and increasing susceptibility to respiratory pathogens.

### 3.6. Serotype Distribution of the Four Major Bacterial Pathogens

#### 3.6.1. Serotypic Diversity of the Four Major Bacterial Pathogens in Clinical Samples

**Figure 9:**
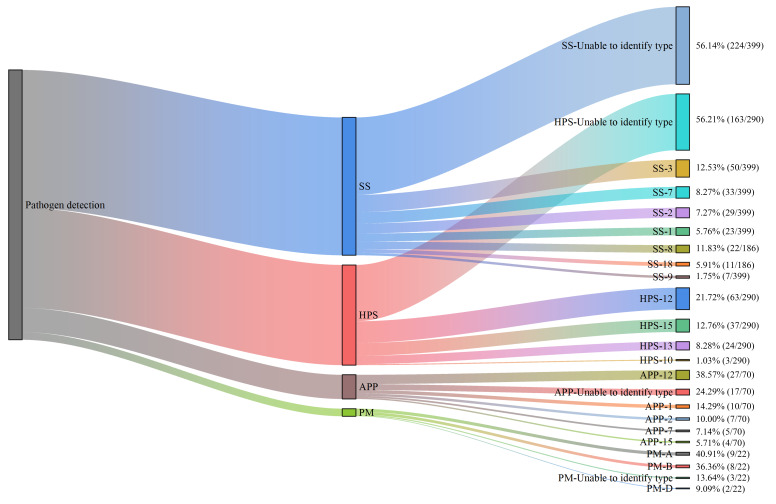
Serotype distribution of HPS, APP, SS, and PM isolates from clinical samples collected in swine farms and an abattoir in Xinjiang. For HPS, serotypes 12 (21.72%) and 15 (12.76%) were predominant among farm samples, with 56.21% untypeable; in abattoir lung samples, serotype 12 was detected in 14.29% (1/7) of positive samples. For APP, serotype 12 (38.57%) dominated in farm samples, followed by serotypes 1 (14.29%), 2 (10.00%), 7 (7.14%), and 15 (5.71%), with 24.29% untypeable. For SS, serotype 3 (12.53%) was most prevalent in farm samples, with 56.14% untypeable; the single SS-positive abattoir sample was serotype 7. For PM, capsular types A (40.91%) and B (36.36%) were predominant in farm samples, followed by type D (9.09%), with 13.64% untypeable; types E and F were not detected.

Positive samples were subjected to PCR-based serotyping targeting common serotypes (Figure 9). Among clinical samples, HPS serotypes 12 (21.72%) and 15 (12.76%) were identified as the predominant serotypes, followed by serotypes 13 (8.28%) and 10 (1.03%), with 56.21% of isolates remaining untypeable. For APP, serotype 12 (38.57%) was the most prevalent, followed by serotypes 1 (14.29%), 2 (10.00%), 15 (5.71%), and 7 (7.14%), while 24.29% of isolates were untypeable. Regarding SS, serotype 3 (12.53%) was the most frequently detected, followed by serotype 8 (11.83%), serotype 7 (8.27%), serotype 2 (7.27%), serotype 18 (5.91%), serotype 1 (5.76%), and serotype 9 (1.75%); notably, 56.14% of SS isolates were untypeable. For PM, capsular types A (40.91%) and B (36.36%) were predominant, followed by D (9.09%), with 13.64% of isolates untypeable. Capsular types E and F were not detected in any PM-positive sample.

In abnormal lung samples collected from slaughtered pigs, HPS serotype 12 was detected in 14.29% (1/7) of positive samples, while the remaining HPS isolates were untypeable. The single SS-positive sample in this set was typed as serotype 7.

#### 3.6.2. Farm-Level Distribution of the Four Major Bacterial Pathogens Serotypes

**Figure 10:**
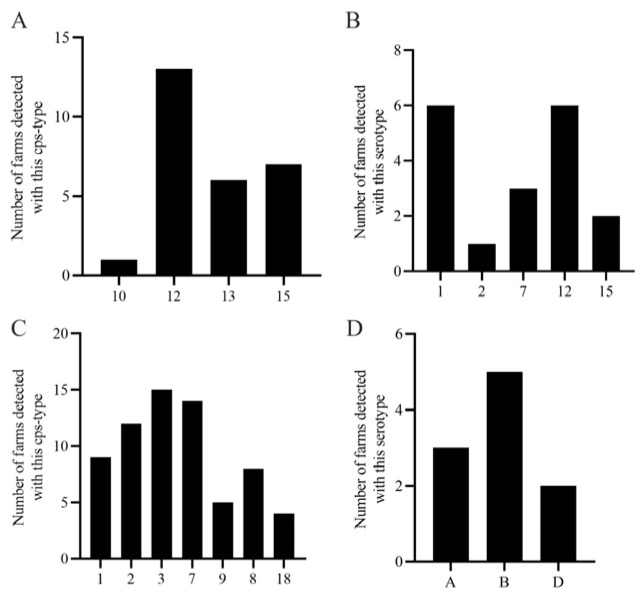
Farm-level distribution of HPS, APP, SS, and PM serotypes across 27 large-scale swine farms in Xinjiang. (**A**) Number of farms in which each HPS serotype was detected. Serotype 12 was the most widely distributed (13 farms), followed by serotypes 15 (7 farms) and 13 (6 farms); serotype 10 was detected in only one farm. (**B**) Number of farms in which each APP serotype was detected. Serotypes 1 and 12 were the most prevalent (each detected in 6 farms), followed by serotypes 7 (3 farms), 15 (2 farms), and 2 (1 farm). (**C**) Number of farms in which each SS serotype was detected. Serotypes 3 and 7 were the most widely distributed (15 and 14 farms, respectively), followed by serotypes 2 (12 farms), 1 (9 farms), 8 (8 farms), 9 (5 farms), and 18 (4 farms). (**D**) Number of farms in which each PM capsular type was detected. Type B was the most widely distributed (5 farms), followed by type A (3 farms) and type D (2 farms).

A total of 27 farms were investigated in this study. Among them, 24 farms tested positive for HPS, with detection rates ranging from 2.25% to 70.59% (Table 1). Across HPS-positive farms, serotype 12 was the most widely distributed, detected in 13 farms, followed by serotype 15 (7 farms) and serotype 13 (6 farms). Serotype 10 was detected in only one farm, confirming serotype 12 as the predominant HPS serotype in this region. Regarding infection patterns, 45.83% (11/24) of HPS-positive farms exhibited mixed-serotype infections, primarily comprising HPS-(12 + 13), HPS-(12 + 15), and HPS-(12 + 13 + 15) combinations. Single-serotype infections were observed in only two farms, both of which were serotype 12 (Figure 10A).

For APP, 16 out of 27 farms tested positive, with detection rates ranging from 1.67% to 29.41%. Serotypes 1 and 12 were the most widely distributed, each detected in six farms, followed by serotype 7 (3 farms), serotype 15 (2 farms), and serotype 2 (1 farm), indicating that serotypes 1 and 12 are the predominant APP serotypes in large-scale swine farms in this region. Among APP-positive farms, single-serotype infections included APP-12 (2 farms) and APP-1 (1 farm); dual-serotype infections included APP-(1 + 7) and APP-(1 + 12) (each in 2 farms), as well as APP-(7 + 12) and APP-(1 + 15) (each in 1 farm); a triple-serotype infection, APP-(1 + 12 + 15), was observed in one farm (Figure 10B).

For SS, the farm-level positivity rate was 89.66% (25/27), with detection rates ranging from 4.08% to 70.97% (Table 1). Within the 25 SS-positive farms (Figure 10C), serotypes 3 and 7 were the most widely distributed, detected in 15 and 14 farms, respectively, followed by serotype 2 (12 farms), serotype 1 (9 farms), serotype 8 (8 farms), serotype 9 (5 farms), and serotype 18 (4 farms). Notably, farms No. 4 and No. 8 harbored seven different serotypes, while farms No. 10 and No. 17 harbored six serotypes. Mixed infections involving three serotypes were observed in 29.63% (8/27) of farms.

For PM, the farm-level positivity rate was 37.04% (10/27), with detection rates ranging from 1.43% to 7.69%. Among PM-positive farms (Figure 10D), capsular type B was the most widely distributed, detected in five farms, followed by type A (3 farms) and type D (2 farms). Infection patterns in PM-positive farms included single infections (PM-B in farms No. 5 and No. 19; PM-A in farm No. 28), dual infections (PM-(A + B) in farm No. 6; PM-(D + B) in farm No. 11), and a triple infection (PM-(A + B+D) in farm No. 9).

### 3.7. Detection of Pathogens in Farm Environment and Personnel Samples

HPS, APP, and PM were not detected in any environmental or personnel samples. However, as an important zoonotic pathogen, SS poses a potential threat to human health, with ten serotypes capable of infecting humans having been identified to date. At the farm level, the detection rate of SS was 73.33% (11/15). Among the eight nasal swabs collected from farm workers, the detection rate was 25.00% (2/8). Additionally, SS was detected in environmental samples collected from cleaning and disinfection stations prior to farm entry, with a detection rate of 21.05% (4/19). In contrast, no SS was detected in samples collected from the procurement department located in the same geographical area (0/6). These findings suggest that SS is present in farm environments and among farm personnel, highlighting potential occupational exposure risks and the need for enhanced biosecurity measures.

## 4. Discussion

Bacterial pathogens associated with PRDC frequently act as secondary or co-infecting agents, exacerbating respiratory damage and leading to conditions such as pneumonia and pleurisy. In recent years, the implementation of antibiotic ban policies has been associated with an increased prevalence of bacterial diseases, with high rates of co-infection posing significant challenges for disease prevention and control [6]. The primary bacterial pathogens implicated in PRDC include HPS, APP, SS, and PM [25]. This study provides a detailed description of the prevalence and serotype distribution of these four pathogens in intensive pig farms in Xinjiang, China, from October 2024 to May 2025. Additionally, as a zoonotic pathogen, the carriage status of SS in farm environments and among farm workers was also investigated.

HPS is traditionally considered a commensal bacterium of the upper respiratory tract in pigs; however, under specific conditions, it can invade the host and cause severe systemic disease. Bello-Ortí [26] demonstrated that non-virulent strains isolated from the nasal cavities of healthy pigs form more substantial biofilms than virulent strains isolated from lesions of diseased pigs, suggesting that biofilm formation may facilitate colonization and persistent carriage of non-virulent strains in the porcine upper respiratory tract. In contrast, the planktonic state of virulent strains may enable their dissemination within the host. In the present study, the detection rate of HPS in nasal swabs (49.26%) was considerably higher than that in tissue samples (10.11%), further corroborating its role as a commensal organism of the porcine upper respiratory tract.

Highly virulent serotypes of HPS, including serotypes 1, 5, 10, 12, 13, and 14, typically induce severe infection and rapid mortality; serotypes 2, 4, 8, and 15 exhibit moderate virulence, whereas serotypes 3, 6, 7, 9, and 11 have been demonstrated to be non-virulent [27]. In this investigation, the overall detection rate of HPS was 22.84%, which is lower than the 27.8% prevalence reported by Ni [28] in Chinese swine herds between 2005 and 2019, and also lower than the 38.55% detection rate documented by Chen [24] in 2022 across 651 samples from 19 farms in Xinjiang.

Regarding serotype distribution, Dong [29] reported that the predominant HPS serotypes in China during 2020–2021 were, in descending order, serotypes 12, 4, 5, and 13, with serotype 12 exhibiting a 28% increase and emerging as the dominant circulating serotype. Our findings are consistent with these observations, as serotype 12 was detected in 48.15% of the farms investigated, confirming that serotype 12 is the prevalent HPS serotype in porcine herds across parts of Xinjiang. HPS serotype 12 is classified as a highly virulent serotype and is unable to form biofilms on polystyrene surfaces, suggesting it may preferentially exist in a planktonic state that facilitates rapid dissemination [30]. Given that serotype 12 has become increasingly prominent in recent years, the development of a serotype 12-based vaccine is warranted for effective HPS prevention and control. Additionally, our survey identified the presence of highly virulent serotypes 10 and 13, as well as the moderately virulent serotype 15. In Chen’s [24] serotyping analysis of HPS-positive samples, serotype 12 was also identified as the predominant type; however, serotype 13 was not detected, suggesting that serotype 13 may have been introduced and disseminated throughout the Xinjiang region during the intervening period.

APP is the causative agent of porcine pleuropneumonia (PCP), a disease with worldwide prevalence. Pigs of all ages are susceptible to APP infection, and in acute cases, affected pigs may succumb within hours. In the present study, the detection rate of APP in tissue samples (12.30%) was substantially higher than that in nasal swabs (0.49%), indicating that APP has low colonization capacity in the nasal cavity but exhibits high pathogenicity once established in the lower respiratory tract. The occurrence of PCP is also associated with factors such as housing conditions, stocking density, and environmental management, and the predominant serotypes vary across different countries and regions. To date, 19 serotypes of APP have been identified, and the weak cross-immunity among them complicates disease control [31]. In Korean pig herds, APP serotypes 1 and 5 have been reported as predominant [32]. In this investigation, the overall detection rate of APP was lower than those of HPS and SS, with serotypes 12 and 1 identified as the predominant circulating serotypes. Additionally, serotypes 2, 7, and 15 were also detected. APP serotype 12 is traditionally considered a low-virulence serotype, while both serotype 12 and 1 strains possess a complete flp (fimbrial low-molecular-weight protein) promoter structure that facilitates upper respiratory tract colonization [33]; this may explain their widespread distribution. In Chen’s 2022 [24] serotyping study of APP-positive samples from Xinjiang, serotype 15 was reported as the dominant serotype; however, serotypes 2, 5, and 7 were not detected, and serotype 12 was not investigated. These findings suggest the emergence of new APP serotypes in the Xinjiang region, highlighting the need for continuous surveillance and updated vaccine formulations.

SS exhibits considerable resistance to various environmental conditions, with its survival time varying significantly under different temperatures. According to the literature, at 60 °C, SS survives for 10 min; at 50 °C, it can survive for up to 2 h; while at low temperatures such as 10 °C, for instance in animal carcasses, SS can persist for as long as 6 weeks. This environmental resilience contributes to its potential for zoonotic transmission. Among the four pathogens investigated in this study, SS exhibited the highest detection rate (32.12%) and was nearly ubiquitous across farms (89.66%). Furthermore, its extensive serotypic diversity and the widespread occurrence of multi-serotype co-infections within single farms pose significant challenges for the prevention and control of SS-associated diseases.

The detection rate of SS was higher in nasal swabs (59.61%) and saliva samples (16.52%) compared to tissue samples (10.93%), further confirming the tonsils and upper respiratory tract as its primary colonization sites [34]. Notably, this pathogen was also frequently detected in farm environmental samples (55.21% of farm samples), whereas its detection rate in abattoir samples was lower. This suggests the existence of potential persistent environmental reservoirs and identifies possible occupational exposure or cross-contamination risk points. SS serotype 3, which was most frequently detected in our study, has been reported to account for approximately 15% of SS clinical cases globally, reflecting its increasing clinical significance [35]. Of particular public health concern, SS was detected in 25.00% (2/8) of nasal swabs collected from farm workers and in 21.05% (4/19) of samples from cleaning and disinfection stations. These findings underscore the occupational health risks associated with SS and emphasize the need for enhanced biosecurity measures and personal protective equipment for farm personnel.

Respiratory disease caused by PM primarily manifests as rhinitis of the upper respiratory tract and pneumonia of the lower respiratory tract. The severity of clinical signs depends on the host immune status and the virulence of the infecting strain; severe cases can progress from pneumonia to fatal septicemia. Within the surveyed region of this study, the detection rate of PM (1.78%) was lower than those of HPS, APP, and SS, indicating a relatively minor impact of PM on large-scale swine farms in Xinjiang. PM was detected exclusively in tissue samples and was absent from both nasal swabs and saliva samples. The identified capsular types were A, B, and D, with type A being predominant; types E and F were not detected. This finding further corroborates that porcine pneumonic lesions caused by PM infection are predominantly associated with capsular type A strains. The hyaluronic acid capsule of serotype A mediates adhesion via CD44 binding, interferes with host signaling, and resists phagocytosis, contributing to weak immunogenicity [36]. The limited serotypic diversity and low detection rate suggest that PM may be less of a priority for immediate intervention compared to the other three pathogens in this region.

In summary, the epidemiological characteristics of PRDC-associated bacterial pathogens in Xinjiang are as follows: serotype replacement in HPS (with serotype 12 emerging as dominant), emergence of new serotypes in APP (serotypes 12 and 1), widespread environmental contamination with high serotypic complexity in SS, and limited dissemination of PM. It is recommended that future prevention and control strategies focus on developing or optimizing multivalent vaccines targeting the predominant serotypes identified in this study, implementing precision immunization protocols guided by regular serotype, and emphasizing environmental disinfection and improved husbandry practices to mitigate the risks of co-infection and cross-contamination. The detection of SS in farm workers highlights an often-overlooked occupational health dimension that warrants further investigation and intervention. This study fills a critical gap in systematic epidemiological data on PRDC-associated bacterial pathogens in Xinjiang in recent years, providing a scientific basis for the formulation of regionally tailored precision prevention and control strategies, as well as for subsequent vaccine development.

## 5. Conclusions

This study provides a comprehensive epidemiological characterization of four major bacterial pathogens associated with PRDC in large-scale swine farms in Xinjiang. The findings reveal distinct serotype profiles and high co-infection rates, with SS and HPS emerging as the predominant pathogens, and SS posing a notable zoonotic risk through environmental and occupational exposure. These results underscore the need for regionally tailored intervention strategies grounded in the One Health approach, including multivalent vaccines targeting locally prevalent serotypes, antimicrobial stewardship informed by regional susceptibility data, strengthened biosecurity measures, and integration of molecular surveillance tools such as whole-genome sequencing. Given the single-region scope and limited time frame, the generalizability of our findings is constrained, and further work incorporating genomic epidemiology and antimicrobial resistance profiling across broader geographic areas will be essential to refine prevention strategies. Collectively, these efforts will help mitigate economic losses, curb antimicrobial resistance, and protect both animal and public health.

## Figures and Tables

**Table 1:** Detection rates and serotype profiles of HPS, APP, SS, and PM in different large-scale swine farms.

Farm (No.)	HPS		APP		SS		PM	
	Detection Rate	Serotypes Present	Detection Rate	Serotypes Present	Detection Rate	Serotypes Present	Detection Rate	Serotypes Present
1	29.63%	12, 13, 15	0%	-	55.55%	1, 2, 3, 7	0%	-
2	5.00%	12, 13	0%	-	29.00%	1, 2, 3, 7, 8	0%	-
3	0%	-	4.08%	\	4.08%	3	0%	-
4	32.26%	12, 13, 15	9.68%	\	70.97%	1, 2, 3, 7, 8, 9, 18	0%	-
5	8.57%	12, 15	14.29%	1, 12	40.00%	1, 3, 7	1.43%	B
6	3.45%	\	1.72%	\	12.07%	\	17.24%	A, B
7	6.98%	\	6.98%	\	6.98%	2	0%	-
8	21.05%	12, 15	0%	-	42.11%	1, 2, 3, 7, 8, 9, 18	1.75%	\
9	35.29%	12, 13	8.82%	1, 7	41.18%	1, 9	5.88%	A, B, D
10	26.23%	10, 12, 13	6.56%	7, 12	57.38%	1, 2, 3, 7, 8, 9	1.64%	\
11	29.63%	12, 15	22.22%	12	18.52%	2, 3, 7	3.70%	B, D
12	22.77%	\	2.97%	1, 12, 15	6.67%	\	0%	-
13	5.88%	\	29.41%	1, 12	25.49%	3, 7, 8	1.96%	\
14	40.00%	12	0%	-	60.00%	1, 3, 7	5.00%	\
15	32.5%	12, 13	2.5%	12	25%	3, 7, 9	0%	-
16	2.94%	\	0%	-	14.71%	\	0%	-
17	65.96%	12, 15	4.26%	1, 15	61.7%	1, 2, 3, 7, 8, 18	0%	-
18	0%	-	8.82%	1, 7	0%	-	0%	-
19	11.54%	\	0%	-	11.54%	\	3.85%	B
20	0%	-	0%	-	0%	\	0%	-
21	70.00%	\	1.67%	\	85.00%	\	0%	-
22	23.81%	12	0%	-	28.57%	2, 3, 7	0%	-
23	10.00%	\	0%	-	70.00%	2, 3, 7	0%	-
24	2.25%	\	0%	-	21.35%	2, 7	0%	-
25	40.91%	\	0%	-	9.10%	18	0%	-
26	23.08%	\	7.69%	\	7.69%	18	7.69%	A
27	70.59%	12, 15	11.76%	1	47.06%	2, 3, 8	0%	-

‘-‘ indicates that the pathogen was not detected in this farm, and therefore serotyping was not performed; ‘\’ indicates that the pathogen was detected in this farm, but none of the targeted serotypes were identified.

## Data Availability

The original contributions presented in this study are included in the article. Further inquiries can be directed to the corresponding authors.

## Abbreviations

The following abbreviations are used in this manuscript:

PRDC	Porcine Respiratory Disease Complex
HPS	Glaesserella parasuis
APP	Actinobacillus pleuropneumoniae
SS	*Streptococcus suis*
PM	Pasteurella multocida
PCP	porcine pleuropneumonia
AI	artificial intelligence

## Appendix A

**Table A1:** Primer sequences for species identification and serotyping of HPS, APP, SS, and PM.

Gene Name	Primer Sequence (5′-3′)	Fragment Size (bp)	Annealing Temperature (°C)
HPS	ACAACCTGCAAGTACTTATCGGGAT	275	59
	TAGCCTCCTGTCTGATATTCCCACG		
HPS-10	GGTGACATTTATGGGCGAGTAAGTC	790	59
	GCACTGTCATCAATAACAATCTTAAGACG		
HPS-12	ATGGCTCACGATCCGAAAG	508	56
	ATTTCCCTTTCCTAAACGC		
HPS-13	GCTGGAGGAGTTGAAAGAGTTGTTAC	840	59
	CAATCAAATGAAACAACAGGAAGC		
HPS-15	CAAGTTCGGATTGGGAGCATATATC	550	59
	CCTATATCATTTGTTGGATGTACG		
APP	TTATCCGAACTTTGGTTTAGCC	418	59
	CATATTTGATAAAACCATCCGTC		
APP-1	CTGGAGTAATTACGGCGACTATTCC	959	56
	AGGAGAAGCTAGTAGTACTTGCATTTTC		
APP-2	GAGTGTGATGATGATGCTCTGGTTC	247	56
	TACCAATAACTGTTGCAACTAACGC		
APP-7	TTGGAATGGATTCATGATTGGGC	601	56
	CGGAAATGGCCTATTGAAAAACG		
APP-12	TAAAGGTATTATAACGCCGGCTCT	347	56
	CTCCCATCTGTTGTCTAAGTAGTAG		
APP-15	GCAACTTGGAGAACATGGTTAAATCAAG	1595	56
	CAACCCTCCAATGTAAGCGAAGG		
SS	GCAGCGTATTCTGTCAAACG	689	56
	CCATGGACAGATAAAGATGG		
SS-1	TTTAGTAGACGAAAACGGGT	461	56
	TTGGCAAGAACTCATTATCC		
SS-2	TTCGTATTAACTTACTTGGCGT	363	53
	TAAATCCCCATATGCCAAATCC		
SS-3	ACATCCATTGCAGGAGTAGT	210	53
	TGCAGTTCCAAAATTCTTCGT		
SS-7	AAAATTCGTTCCATTGTAGGTG	609	55
	TGAAGTTGAAGCTGGTGATAAA		
SS-8	ATCGCTTCAAATAAGGTAGGAG	268	50
	TGTAGGCCGTAATATCAACAAA		
SS-9	TGAAAGTAGGTATATCTCAGCA	809	55
	AAAGAATTGAATCCCACCTGAG		
SS-18	ATAGGCTGTACTTTGATAACCG	310	55
	AGCCTATCGCTCAAAAACTTAT		
PM	ATCCGCTATTTACCCAGTGG	457	56
	GCTGTAAACGAACTCGCCAC		
PM-(A)	GATGCCAAAATCGCAGTCAG	1048	56
	TGTTGCCATCATTGTCAGTG		
PM-(B)	CATTTATCCAAGCTCCACC	758	56
	GCCCGAGAGTTTCAATCC		
PM-(D)	TTACAAAAGAAAGACTAGGAGCCC	647	56
	CATCTACCCACTCAACCATATCAG		
PM-(E)	TCCGCAGAA AATTATTGACTC	512	56
	GCTTGCTGCTTGATTTTGTC		
PM-(F)	TCGGAGAACGCAGAAATCAG	852	56
	TTCCGCCGTCAATTACTCTG		

**Table A2:** Summary of clinical and environmental sample collection.

Survey Location	Pig Farm (No.)	Clinical sample collection					Environmental or Personnel-Related Samples from Aquaculture Settings
		Tissue Sample	Nasal Swabs	Saliva Samples	Clinical Abnormalities	Total	
Changji City	1	-	-	2	25	27	8
Changji City	2	20	26	19	35	100	17
Manas County	3	-	-	39	10	49	-
Hutubi County	4	10	-	-	29	39	5
Wujiaqu	5	29	-	-	41	70	21
Huyanghe City	6	56	-	-	2	58	-
Huyanghe City	7	39	-	-	4	43	-
Wujiaqu	8	3	-	-	54	57	15
Wujiaqu	9	-	-	18	16	34	8
Changji City	10	2	-	23	36	61	-
Shawan City	11	40	6	-	8	54	-
Hutubi County	12	56	20	14	11	101	5
Hutubi County	13	51	-	-	-	51	-
Hutubi County	14	-	4	-	16	20	-
Manas County	15	1	16	-	23	40	1
Manas County	16	2	-	-	32	34	3
Wusu City	17	5	75	-	14	94	2
Shihezi City	18	2	-	-	32	34	-
Shihezi City	19	-	-	-	26	26	-
Shawan City	20	1	-	-	1	2	-
Manas County	21	7	45	-	8	60	-
Manas County	22	10	11	-	-	21	6
Wusu City	23	4	-	-	6	10	-
Hutubi County	24	1	-	-	88	89	6
Fukang City	25	-	-	-	22	22	-
Fukang City	26	-	-	-	26	26	5
Miquan City	27	2	-	-	15	17	2
Abattoir (Wujiaqu)	28	25	-	-	-	25	-
	Total	366	203	115	580	1264	
Disinfection and isolation of dormitories	29						19
Procurement Department	30						6
	SUM						129

Note: “-” indicates “no samples of this type were collected”.
